# Impact Resistance of Polypropylene Fibre-Reinforced Alkali–Activated Copper Slag Concrete

**DOI:** 10.3390/ma14247735

**Published:** 2021-12-15

**Authors:** Vijayaprabha Chakrawarthi, Siva Avudaiappan, Mugahed Amran, Brindha Dharmar, Leon Raj Jesuarulraj, Roman Fediuk, Radhamanohar Aepuru, Nikolai Ivanovich Vatin, Erick Saavedra Flores

**Affiliations:** 1 Department of Civil Engineering, Alagappa Chettiar Government College of Engineering and Technology, Karaikudi 630001, India; 2 Departamento de Ingeniería en Obras Civiles, Universidad de Santiago de Chile, Av. Ecuador 3659, Estación Central, Santiago 8330378, Chile; siva.avudaiappan@usach.cl (S.A.); erick.saavedra@usach.cl (E.S.F.); 3 Department of Civil Engineering, College of Engineering, Prince Sattam Bin Abdulaziz University, Alkharj 16273, Saudi Arabia; 4 Department of Civil Engineering, Faculty of Engineering and IT, Amran University, Amran 9677, Yemen; 5 Department of Civil Engineering, Thiagarajar College of Engineering, Madurai 625015, India; dbciv@tce.edu; 6 Applied Civil Engineering Group, CSIR-North East Institude of Science and Technology, Jorhat 785006, India; leonraj@neist.res.in; 7 Polytechnic Institute, Far Eastern Federal University, 690922 Vladivostok, Russia; fedyuk.rs@dvfu.ru; 8 Departamento de Ingeniería Mecánica, Facultad de Ingeniería, Universidad Tecnologica Metropolitana, Santiago 8330378, Chile; raepuru@utem.cl; 9 World-Class Research Center for Advanced Digital Technologies, Peter the Great St. Petersburg Polytechnic University, 195251 St. Petersburg, Russia; vatin@mail.ru

**Keywords:** impact resistance, alkali-activated copper slag, polypropylene fibre, waste replacement

## Abstract

Copper slag (CS) is produced during the smelting process to separate copper from copper ore. The object of the experimental research is to find the optimum percentage of CS and PPF volume fraction when CS replaces fine aggregate, and PPF volume fraction when subjected to impact loading. Copper slag was incorporated as 20%, 40%, 60%, 80% and 100% with PPF of 0.2–0.8% with 0.2% increment. The number of blows on failure of the specimen increases as the fibre volume increases. In addition, the energy absorption of composite concrete is higher than that of ordinary concrete. Concrete with up to 40% CS and 0.6% PPF volume shows a 111.72% increase in the number of blows for failure as compared to the control specimen. The impact resistance at failure was predicted by regression analysis, and very high regression coefficients of 0.93, 0.98 and 0.98 were obtained respectively at 7-, 14- and 28-days curing. In addition to regression analysis, a two-parameter Weibull distribution analysis was used to obtain reliable data on the number of blows at first cracking and eventual failure. The energy absorption at 28-day curing period is 1485.81 Nm which is 284% higher than the control mix. Based on the findings, it can be inferred that adding CS up to 60% densifies the microstructure due to its pozzolanic activity, while polypropylene fibre acts as a micro reinforcement, increasing the number of blows.

## 1. Introduction

Cement mortar concrete is widely acknowledged as the most extensively employed construction material on the globe. The advancement in infrastructure in the world has accelerated the use of concrete [1]. Large quantities of natural resources are consumed in the manufacturing process of concrete. Approximately 60–80 percent of the volume of concrete is made up of aggregates. Natural sand is one of the non-renewable resources used as fine aggregate, which is depleting tremendously [2,3,4].To reduce the consumption and to protect natural resources for future generations, it is an urgency to use non-conventional and renewable materials in concrete production [5,6]. The world is on the lookout for new material as fine aggregate for concrete production. However, due to the rapid industrial revolution, waste disposal is becoming more complex, posing the challenge of recycling and reusing waste generated [7].

Researchers are attempting trials to recycle, reduce and reuse the industrial wastes in different ways such as construction material or by developing new value-added products [8,9,10]. Copper slag is one such waste by-product produced from the copper smelting process in copper-mining industries. For each tonne of copper produced, 1.2 tonne of copper slag is created. Copper slag has many recycling utilizations such as metal recovery, production of value-added products, bonded abrasive tools, roofing copper granules, cutting tools, abrasive cleaning tiles, glass, road pavement base material, track ballast, asphalt mix for pavements, etc. A larger self-weight and more significant overburden pressure finds application for it in soil stabilization [11]. Copper slag has more dimensional stability, which will improve impact resistance [12]. From previous literatures it is noted that the compressive strength was comparable or higher in all mixes with various copper slag concentrations [13]. With up to 50% copper slag, the compressive strength of cement mortars increased by 70%. Fresh concrete’s workability improves considerably as the copper slag percentage rises [14]. The concrete density increases by 5% as a result of its high self-weight. In addition, 40–50% copper slag showed comparable strength with control concrete [15]. Copper slag has a low water absorption rate, which helps it gain strength in concrete. As a result, several researchers indicate that a replacement percentage of CS in the range of 40–50% meets the strength and durability criteria [16].

The microstructure studies of CS explored that silica, alumina, lime and magnesia are available in copper slag. The oxides of silica-alumina, ferrous and magnesium make up 95% of the total mass of chemical constituents [17]. The addition of CS induces the pozzolanic reaction during hydration. In conventional concrete, some calcium hydroxide molecules would not participate in the hydration process. In the presence of CS, the unreacted crystalline (Ca(CO_3_)) in control concrete was converted into a calcium-silicate-hydrate reacted component [17,18]. The microstructure of copper slag concrete discloses that the grains are not rounded up with water molecules. The grains are surrounded up by fully hydrated particles that conform to the pozzolanic behaviour of CS. The hydration product shows the basic patterns of Fe_3_O_4_ (magnetite) and Fe_2_SiO_4_ (fayalite) as the primary phases in XRD analysis. Hydration is measured by the formation of a calcium hydroxide peak in the control concrete. A slight increase in the calcium hydroxide peaks for 10% replacement of CS is observed compared to ordinary concrete [19].

Cracking is a critical parameter that predominantly affects the durability, structural integrity, bonding between binder and fillers and load bearing capacity. Even the micro-cracks are the pathfinder for making wider cracks [20]. Fibre-reinforced concrete (FRC) was discovered, which acts as a micro reinforcement that prevents formation of micro-cracks, improves tensile strength, modulus of elasticity, energy absorption capacity and ductility of the concrete [21]. Fibres perform well in reducing plastic shrinkage cracks, minimizing thermal cracks and enhancing impact resistance, etc. [22,23,24,25]. FRC is a type of concrete made up of short, discontinuous fibres that are uniformly dispersed and oriented randomly in the concrete matrix [26]. The properties of fibre reinforced concrete depend on composition of fibre, volume fraction of fibre, slenderness ratio, distribution of fibres and fibre orientation. Depending on the geometry of the fibres and the type of application, the volume of fibres admixed to conventional concrete varies from 0.2 to 1.5% [27,28]. One of the past studies employing fibre reinforced concrete in sleepers revealed that 1–1.5% of PPF showed slight influence on density, and twisted PPF ensures uniform dispersion and improved performance [29]. Fibre-reinforced polymer incorporated into concrete to increase the ultimate load, ductility and energy absorption capacity [30]. Polypropylene is a type of synthetic fibre made from monomeric H_3_C_6_ hydrocarbon polymer. The polypropylene fibres are classified as monofilament and fibrillated fibre. Fibrillated fibres are made by forming slits in a plastic film having micron thickness. Fibrillated fibres are stretched to a particular length making a fibre bridging phenomenon that improves the hardened characteristics of concrete [31,32]. Hybrid fibres or combination of two different fibres incorporated in reinforced concrete beams show 1.6 times higher energy absorption and improves ductility [33]. 

Plain concrete is a heterogeneous material, and it does not sustain the sudden or time dependent loading such as impact and dynamic loading. The structure’s response to the dynamic loading becomes essential when forces like an earthquake, wind force, fluid sloshing and heavy machinery vibration act. An impulsive loading is possible in constructing the foundation for heavy machinery and circumstances like pile driving for the particular case of foundations [34]. These forces will cause severe damage to the structure and subsequently life losses. There are many such structures or parts of structures that have to counteract the impact loading. Aggregates are tested for their impact strength while used for pavement structures. CS with high specific gravity could withstand the impulse loading as well as its dimension. Therefore, a new composite material developed from CS and PPF is employed to check its impact resistance when incorporated into the concrete mixture.

The novelty of this research work is finding an alternate material for fine aggregate in concrete making. The new material should be waste material from industry also disposed of or dumped in open land; because waste utilization in concrete leads to a sustainable environment. Copper slag is an environmental pollutant discharged or dumped in open ground by Sterlite Industries that induces pozzolanic property when mixed with cement. Incorporating copper slag in concrete improves the mechanical property of concrete. At the same time, the depletion of natural resources like river sand leads to a search for an alternate material to fine aggregate in concrete. Hence the applicability of copper slag as a fine aggregate is verified and the impact resistance of concrete was evaluated. Copper slag available in a similar size fraction as river sand can be used as an alternate material to river sand. It is the reason to replace the copper slag with fine aggregate. To improve the inherent properties, reduce crack propagation and reduce the shrinkage effect, PPF was selected to incorporate concrete along with CS. Past literature was found in the analysis of various mechanical and durability characteristics of copper slag concrete in combination with other waste materials. However, there is no detailed investigation involved in analysing the effect of replacement of CS to river sand in fibre reinforced concrete. This research extends the analysis of copper slag concrete admixed with fibrillated polypropylene fibre subjected to impact loading. The impact resistance of copper slag concrete admixed with polypropylene fibre is the main focus of this research. The main objective of the study is to obtain an optimum replacement of copper slag and optimum volume fraction of polypropylene fibre. The research question of the study is how the CS and PPF combinedly participate in forming a dense microstructure to resist the impact loading and what the optimum replacement of CS is and how much PPF needs to be admixed? Mechanical properties, such as compressive strength and tensile strength of CS-PPF concrete is analysed and compared with control mix. Microstructure of the control concrete and CS-PPF concrete is also compared. 

## 2. Research Significance

That scarcity of natural river sand amplified the research on the feasibility of CS in concrete production. Sterlite Industries India Ltd. Thoothukudi, India, Tuticorin is facing a huge problem disposing of the copper slag into the environment without proper treatment. The plant produces around 4 million tons of copper slag per year. The chemical composition, particle size distribution and low water absorption are CS’s potential beneficial characteristics, enhancing mechanical and durability properties. The chemical composition revealed that the copper slag contains enough oxygen components to act as a natural pozzolan. Hence, the research was extended to incorporate some admixture-like fibres to upgrade the performance of concrete. Fibrillated PPF was added to the CS concrete to improve the intrinsic characteristics such as tensile strength, modulus of elasticity, impact and toughness of concrete to reduce shrinkage cracks. There is still lack of studies available on 100% replacement of CS replacement with PPF on impact resistance of copper slag concrete. 

## 3. Experimental Procedure 

### 3.1. Materials 

Chettinad ordinary Portland cement of OPC 43 grade conforming to BIS 8112, 2013 [35] was used. Cement has specific gravity of 3.14, specific surface area of 260 m^2^/kg and 0.60% residue on 90 µm sieve IS1727 [36]. The test on setting of cement reveals 36% standard consistency [37], 35 minutes of initial setting time and 420 minutes of final setting time [38]. Conplast SP430, a naphthalene-based superplasticizer, was employed at a dose of 0.5% weight of cement material, in accordance with [39]. It has a specific gravity of 1.235, pH 8.5 ± 0.5 and 20 ± 6 poise viscosity. Natural river sand of 1380 kg/m^3^ bulk density, 1.25% water absorption, specific gravity of 2.51 and fineness modulus of 2.74 were used. The grading zone of fine aggregate is zone II in which 79% of mass is presented between 0.15 and 1.18 mm mesh sieves. Copper slag was obtained from the open disposal at Sterlite Corporation of India, Tuticorin. It has specific gravity of 3.56, of bulk density of 2088 kg/m^3^, 2.99 fineness modulus and 0.15% water absorption. The specific gravity of CS is 41.8% greater and CS absorbs 88% less water than river sand. Leaching of heavy metals in copper slag are within allowable limits. Regarding, heavy metal leaching, the study of copper slag revealed that copper slag leaches 0.0368 ppm withing 24 h, and by the end of 180 days it was 0.668 ppm. Both the values are under permissible limits according to TCLP (toxic characteristic leaching procedure) test [40]. U.S. Environmental Protection Agency (USEPA) reported in 1990 that the copper slags from mineral processing are not hazardous [41]. Hence the utilization of copper slag in concrete does not cause any negative impacts to the surroundings. The unoccupied moisture content gets accumulated and accelerates the hydration process. The concrete made with CS enables high fluidity due to low water absorption and glassy texture and can adopt a lower water-cement ratio for the mix design [42,43]. Coarse aggregates were obtained of a crushed angular type with a 20 mm nominal size, having specific gravity of 2.77, fineness modulus of 6.133 and bulk density of 1420 kg/m^3^. Aggregate was tested according to IS 2386 design code [44]. Fibrillated polypropylene fibre was purchased from Jeetmull Jaichandlal Pvt Ltd, Kolkata, West Bengal, India, Chennai, and used as admixture. It has a density of 0.91 kg/m^3^, 10 to 12 mm graded length, 35–40 µm thickness, and 15–18% elongation conforming to ASTM C-1116 [45].

**Figure 1:**
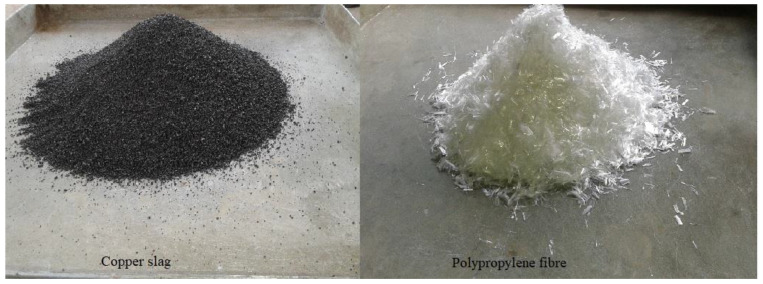
CS and fibrillated PPF.

**Figure 2:**
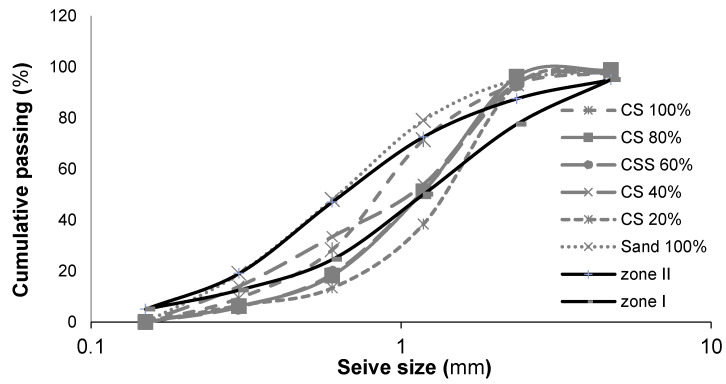
Particle size distribution of fine aggregates.

Table 1 represents the properties of aggregates. Copper slag and fibrillated polypropylene fibre are illustrated in Figure 1. Sieve analysis was done as per [46] codal provision. Figure 2 represents the gradation curve of different percentages of CS replacement. The particle size distribution of CS is similar to that of natural sand in zone II, which is substantiated by earlier research [16]. The chemical constituents of cement and copper slag are compared in Table 2. Copper slag evidences 95.35% of the total composition with aluminium, silica and ferrous as significant components. Both cement and copper slag contain an equal amount of these materials. It is also found that the specification, if the total oxide portion exceeds 75%, the material can produce pozzolanic material [47]. 

### 3.2. Concrete Mix Proportion

The concrete mix proportion was prepared according to BIS 10262, 2019 [48]. Seven parameters have been considered in the mix design, such as cement, coarse aggregate, fine aggregate, water, PPF, CS and super plasticizer. The fine aggregate is categorised into sand and CS. Among these parameters, sand, CS and PPF are considered to be variables. The mix was designed to obtain a characteristic compressive strength of 30 N/mm^2^ (M30 grade). Different mix proportions were arrived on for 20, 40, 60, 80, and 100 percentage of CS replacement. Fibrillated polypropylene fibre was admixed as the percentage of 0.2, 0.4, 0.6 and 0.8 percentage of the volume of concrete. In total, 29 mix proportions were designed, including the control concrete. Mix identification C0P0 is given to control concrete where ‘C’ represents copper slag and ‘P’ represents PPF volume fraction. C denoted from 0 to 100 and P indicated from 1 to 4 for corresponding PPF volume fraction of 0.2–0.8%. The target mean strength of M30 mix is 38.25 N/mm^2^ as per mix design calculation. Table 3 illustrates the mix proportion of the control concrete. Different mix proportions were arrived on by varying river sand, CS and PPF. The fixed cement, water and coarse aggregate content are 363 kg/m^3^, 0.41 kg/m^3^ and 1343 kg/m^3^, respectively. Table 4 represents the overall mix proportions for this experimental work.

### 3.3. Equipment Description and Impact Testing Procedure

**Figure 3:**
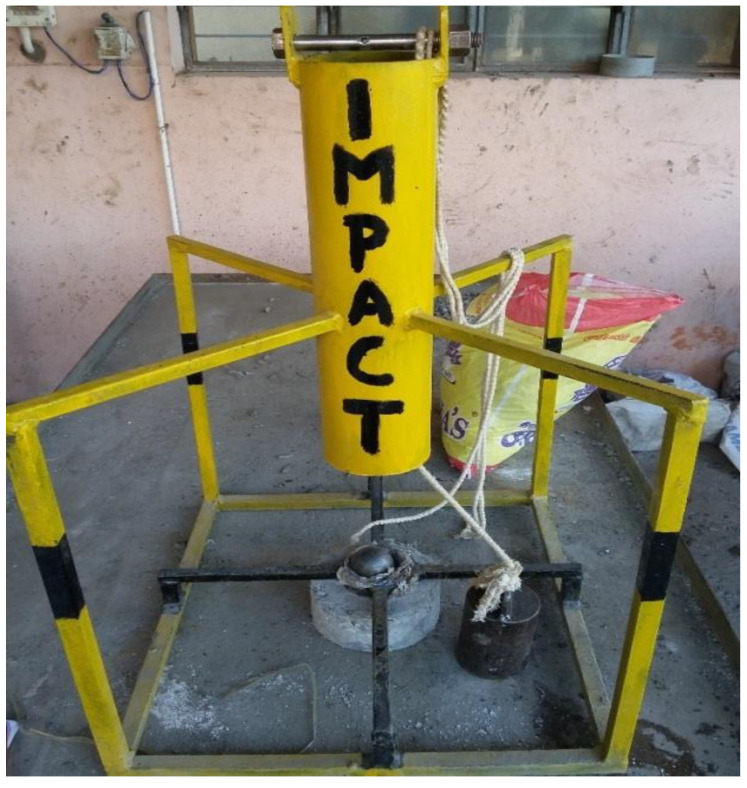
Impact testing machine.

Previous studies have described the methods of fabrication of the impact testing machine [49]. A 34.335 N hammer force was applied as a sudden impact from a predetermined height. A 90 mm diameter, 80 mm high cylindrical ball (the plunger) with a hemispherical blunt tip to a height of 20 mm makes up the test setup. For 25 mm and 30 mm thick slabs, the drop height is taken as 1.185 m and 1.18 m, respectively. Another author [50] performed impact tests with a 3.5 kg hammer with a diameter of 6.4 cm, a length of 30.5 cm, a height of fall of 61.5 cm and a steel ball with a diameter of 6.25 cm and a weight of 0.8 kg. The impact testing machine was fabricated in our laboratory and tested (Figure 3) according to ACI 544 [51] recommendations. It consists of a cylindrical dropping assembly through which the hammer is raised to the required height. A horizontal circular bar with a central hole is fixed on the top of the cylinder to allow the lifting rope through it. The hole in the middle ensures the verticality and freefall when lifting the hammer. The bottom assembly consists of a square frame of 500 mm × 500 mm made of square rods. This frame connects the cylindrical section at the top and the cylindrical sleeve at the bottom. A cylindrical sleeve is placed at the middle of the base assembly using square rods to fix the steel ball. 

The steel ball weighs 1 kg and has a diameter of 65 mm. It arrests the lateral movement of the steel ball. The steel ball transfers the impact load to the specimen. The drop hammer is then placed vertically with its base on the steel ball. The hammer is hoisted and dropped repeatedly to check its verticality and free-fall.

**Figure 4:**
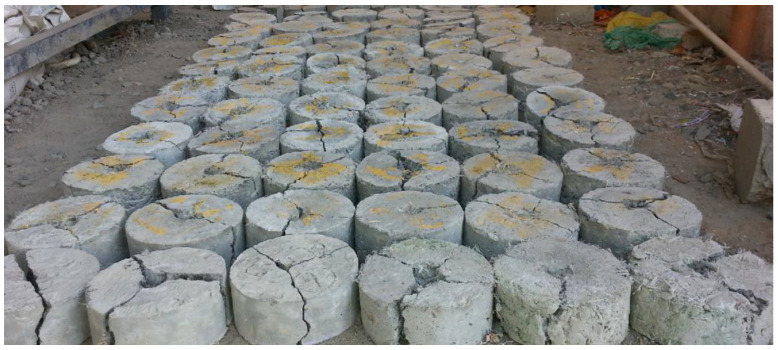
Tested specimens.

The size of the specimen specified by the ACI committee for this study was a circular disc with a diameter of 152 mm and a height of 63.5 mm [52]. The mould is made from polyvinyl chloride pipe of diameter 152.4 mm and thickness 64 mm. The hammer weight is 4.54 kg and the dropping distance is 457 mm. The number of blows for the first apparent break is N1, and the number of blows up to final failure is N2. The identification of the first crack was based on visual inspection. Tested specimens are shown in Figure 4. For each proportion, 9 specimens respectively for 7, 14 and 28 days were cast and in total 270 specimens were tested.

For each specimen, the numbers of blows at first crack (N1) and at ultimate failure (N2) were recorded. The following formula given in Equation (1) was used to compute the impact energy given to each sample:

EI = N × mgh(1)

where, EI—impact energy (N·m), N—the number of blows, m—the mass of the drop hammer (kg), g—gravity acceleration (N/kg) and h—height of drop hammer (m). 

## 4. Results and Discussions 

### 4.1. Compressive Strength of CS-PPF Concrete

**Figure 5:**
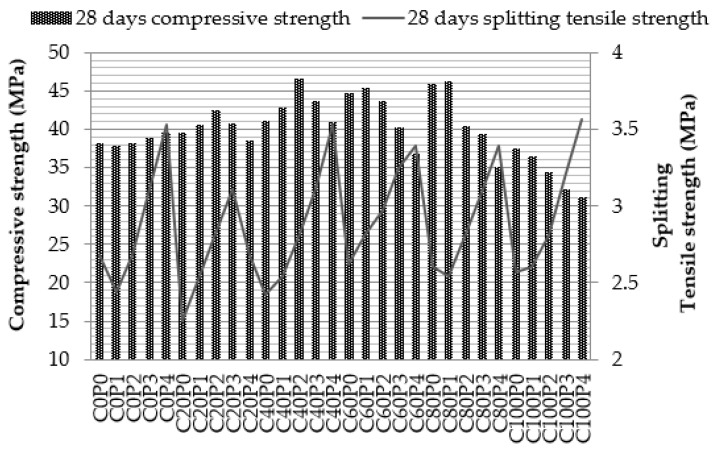
Compressive strength and tensile strength of concrete.

The maximum compressive strength of C0P4 mix at 28 days is 39.60 N/mm^2^, which is 6.55% higher than the strength of the control concrete and 3.5% more than the target mean strength. In Figure 5 the bar chart variation represents the compressive strength of concrete. The maximum compressive strength of C20P2 mix at 28 days is 42.41 N/mm^2^ which is 14% higher than the strength of the control concrete and 10.8% more than the target mean strength. Copper slag 20% contributes 2.54–21.54% increase in compressive strength compared to the control specimen in overall curing periods. The maximum compressive strength of C40P2 mix at 28 days is 46.67 N/mm^2^, which is 25% higher than the strength of the control concrete and 22% more than the target mean strength. The maximum compressive strength of C60P1 concrete at 28 days is 45.48 N/mm^2^, which is 22% higher than the strength of the control concrete and 18.9% more than the target mean strength. The maximum compressive strength of C80P1 concrete at 28 days is 46.22 N/mm^2^, which is 24% higher than the strength of the control concrete and 20.83% more than the target mean strength. The compressive strength of C100P4 concrete at 28 days reduces by up to 16%, the highest reduction observed in this experiment. The decrease in compressive strength is due to the excess copper slag, which dilutes the mix where fibre does not disperse properly. From the existing literature [15], the highest compressive strength was obtained at 40% copper slag and an increasing trend of compressive strength was observed up to 60% of copper slag. Additionally, 100% copper slag replacement substantially reduces the compressive strength of concrete due to excess water content.

### 4.2. Tensile Strength of CS-PPF Concrete

The line graph in Figure 5 shows the variation of tensile strength of concrete. The maximum split tensile strength of C40P4 mix is 3.537 N/mm^2^, which is 31.58% greater than the reference mix. Parveen et al. (2013) [53] reported that the tensile strength of concrete is raised to 47% in fibre reinforced concrete. In overall copper slag replacements, the split tensile strength of concrete increases from 16 to 32% compared to the control mix. In each series of copper slag replacements, PPF volume fraction of 0.8% records the highest split tensile strength. Consequently, the addition of PPF in copper slag concrete enhances the split tensile strength of concrete compared with copper slag alone.

### 4.3. Failure Pattern on Impact Specimens 

**Figure 6:**
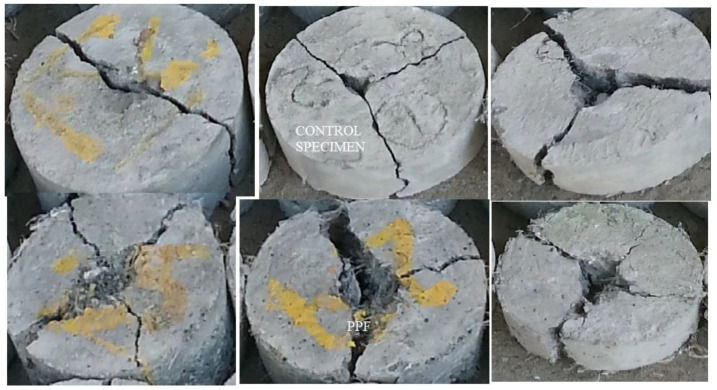
Different failure patterns of the tested specimens.

Different failure patterns of specimens are shown in Figure 6. For one of the specimens, the failure plane divides the sample into two portions. In another specimen, the specimen split into three and especially PPF incorporated samples split into four parts. The failure occurs quickly, and the surface undergoes sudden fracture when broken into two pieces. Small microcracks formed at the surface open up for the macrocracks when it linked with an aggregate. Afterward, it allows ingress of water, and deterioration of concrete quickly starts [50]. 

PPF specimens sustain the impact force and delay sudden fracture and hence some specimens split into four parts. The failure mechanism of CS-PPF concrete is governed by the bridging effect of PPF and improvement in bond strength between cement and fibre. Intrusion of PPF increases the impact resistance and also reduces the anticipated or catastrophic failure of the samples. The fibrillated type of PPF made a networking structure to produce adequate pull-out resistance. The bonding occurs due to the filling effect and pozzolanic activity of CS. The optimum performance was achieved through the dense microstructure and the bridging effect of PPF. The hammer’s concentrated impact force acts as an inertial load on the specimen in the direction of acceleration. The sample attempts to bounce back against the applied load, but the steel ring above stops the movement. The specimens are split into two to four pieces depending upon the stress distribution. The separation of components is due to cracks forming from the point of loading to different radial directions. Fibre admixed specimens do not show complete cleavage along the path of cracks because the cracks are stitched with the fibrillated structure of polypropylene fibre. The short discrete fibres arrest microcracks due to their interconnection in tension region and reduce the friction between the fibres and cement matrix [54].

The fibre reinforced composites exhibit higher resistance to the impact loading than the unreinforced concrete matrix [55]. The crack width in PPF concrete is not nearly as great as in steel fibre reinforced concrete. Fibrillated structures serve as secondary reinforcement in concrete, preventing plastic failure; shrinkage cracks [56].

### 4.4. Impact Resistant at First Crack 

**Figure 7:**
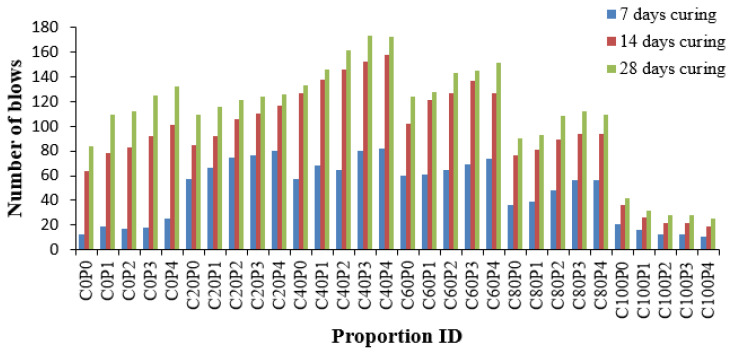
Variation of impact resistance at initial cracking.

The observed values of number of blows are shown in Table 5. The variation of impact resistance for different mix proportions is plotted in Figure 7. The control concrete delivered 84 and 103 blows at initial cracking and ultimate failure of the specimen, respectively. The grade of concrete and w/c ratio is M30 and 0.38, respectively. For the same grade of concrete and 0.48 w/c ratio, one of the studies [57] obtained 37 and 39 blows at first crack and ultimate failure. It is proved that reducing the water/cement ratio increases the impact resistance of concrete. The impact resistance for 0.38 w/c ratio is 1.64 times greater than the mix of 0.48 w/c ratio. Further the number of impact blows increases as the PPF volume fraction is increased [58]. The performance of concrete is unaffected by CS replacement up to 80% as well as the impact strength rises with the volume proportion of PPF. This has been proved by Rahul Sharma [59] that up to 80% the strength and performance is better than control concrete. Specimens with only PPF and no CS exhibit improved first crack and post-first crack performance. Fibrillated polypropylene fibres are dispersed throughout the concrete mix developing an inter cohesion, connectivity and reinforcing effect that leads to a reduction in stress on the surroundings of the micro cracks. The fibre induces a bridging effect and develops homogeneous ductility and post crack energy absorption [25,60].

The number of impacts observed up to the initial crack for the C40P4 concrete mix at 7, 14 and 28 days is 82, 158 and 172, respectively. The control concrete, on the other hand, has 13, 64 and 84. The concrete containing 40% copper slag and 0.8% PPF (C40P4) is highly resistant to initial cracking when subjected to impact loading. The maximum resistance at first crack was 1–5 times higher than the control specimen. 

Impact loading is reduced in concrete containing 100% copper slag. Because CS has finer particles, the particles are crushed when impact loading is applied. Although CS has a high specific gravity, there is much less copper slag in a given volume, necessitating a high cement paste to bind the concrete matrix. Insufficient binding makes the concrete porous and less resistant to applied loading [61]. Since CS has a low water absorption capacity, the quantity of available water increases as the copper slag content rises [62]. Thus, the PPF are clogged, segregated and balled at different places in the concrete. Voids are increased when there is no fibre inside the concrete mix. Hence the number of blows at an initial crack is suddenly reduced below 20 and 40 at 7, 14 and 28 days.

### 4.5. Impact Resistant at Ultimate Failure 

**Figure 8:**
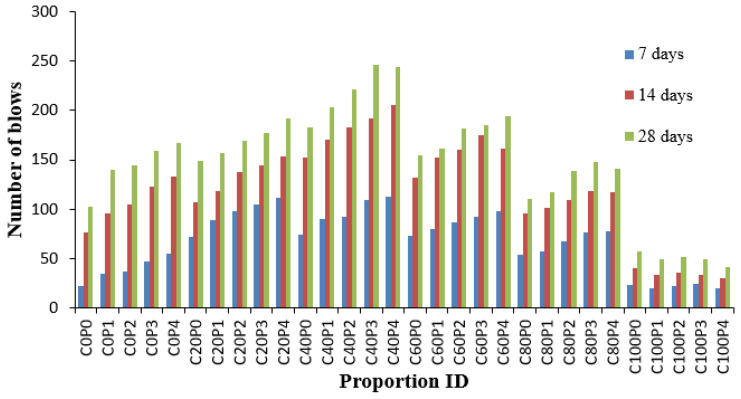
Variation of impact resistance at failure stage.

Figure 8 shows the impact resistance at ultimate failure. The impact blows of C40P3 for failure rose by 111.72% when compared to the control specimen. Because the CS consumes crystalline Ca(OH)_2_ to make additional CSH gel, the microstructure of C40P3 in Figure 6 reveals that more CSH gel is formed in the C40P3 mix. 

Many researchers have proved this theory in research on copper slag [63]. At 28 days of curing for initial crack formation, the number of blows ranges from 25 to 176 in which the number of blows for control concrete is 84. The mean number of blows was 110, with a standard deviation of 41 and a variance coefficient of 375. At 28 days of curing, for the specimen’s ultimate failure the number of blows varies from 42 to 246, whereas the number of blows for the control concrete is 103. The mean value of number of blows was 148, the standard deviation was 55 and the coefficient of variation was 37%. An equal percentage of coefficient variations shows that the point of application of load in both cases is similar in that a rigid metal or a soft mortar might be present [64]. It is concluded that 40% copper slag performs better than any other proportion. Compared to the control specimen, concrete containing 100% copper slag replaced with sand shows a 35–47% reduction in the number of blows required to break the sample. One study [65] found that fibre hybridization improves performance against concrete impact and increases post-cracking strength compared to mono fibre systems.

The blows for the initial crack are increased from 2.66 to 2.91 times at 28 days, while impacts for the post-initial crack increase from 2.75 to 3.10 times. Because of the increased ductility of concrete, the difference between the number of blows at the initial crack and the number of impacts at ultimate failure increases as PPF content increases. One study revealed that for the fibre volume of 0.2%, the number of blows increased to 1.31 times [66]. The study evidenced that 0.25% and 0.6% of PP fibre is the most appropriate proportion for the impact resistance of concrete. This conclusion is in agreement with the results of the present study. 

### 4.6. Microstructural Analysis 

**Figure 9:**
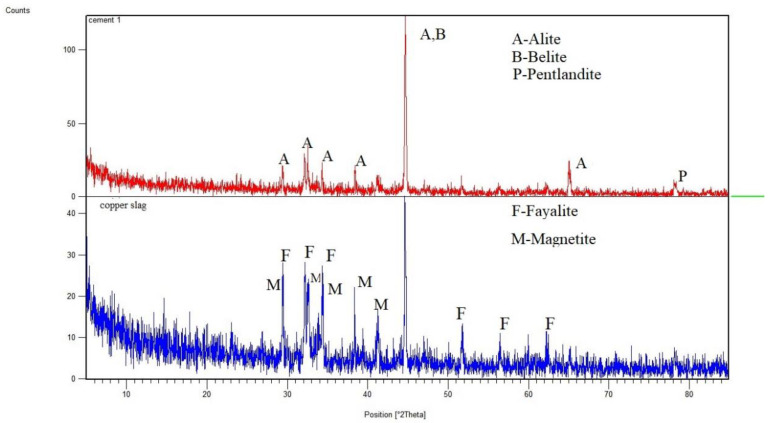
XRD analysis comparison of cement and CS.

The formation of C–S–H gel from dicalcium silicate (belite, C_3_S), tricalcium silicate (alite, C_2_S) and other calcium silicate phases occurs during the hydration process. Figure 9 illustrates the XRD image of cement and copper slag. Cement evidence for alite, belite and pentlandite compounds. The hydration product also includes unreacted calcium hydroxide in crystalline form. Copper slag detects fayalite (Fe_3_O4) and magnetite (Fe_2_SiO_4_) crystalline phase through XRD images [67]. Silicates present in copper slag react with calcium hydroxide to produce extra C–S–H gel, which is homogeneously spread over the entire mass [14]. 

The development of strength in concrete occurs during the hydration process as a result of the condemnatory reaction of tricalcium silicate (C_3_S), which produces C–S–H gel and Ca(OH)_2_, with some Ca(OH)_2_ remaining as an unreacted component. The hydration of tricalcium silicate is a critical reaction in the development of concrete strength (C_3_S). Excess CH (calcium-hydrates) is produced during the hydration of C_3_S and could be consumed by copper slag. More silicate ions (S) are supplied by copper slag, which reacts with CH to form more C–S–H (calcium-silicate-hydrate) gel. Because of the low CaO content, copper slag shows poor cementitious properties. However, when combined with cement, it can produce high pozzolans due to the high concentrations of silica, alumina and iron oxide [68]. The presence of Al_2_O_3_ and SiO_2_ is attributed to critical peaks in the copper slag XRD pattern. Copper slag densifies concrete microstructure due to the pozzolanic activity of silicate ions (SiO_2_) with Ca(OH)_2_. 

The microstructural study revealed that the pozzolanic behaviour of copper slag enhances the hydration reaction. Blended concrete is a denser texture of concrete than the control mix. The dense texture indicates that the cement paste has been tightly bound to the aggregate matrix, and the reacted component has filled the pores between the aggregates. Due to the medium level of pozzolanic activity, some unhydrated Ca(OH)_2_ is unavoidable in CS concrete. The dense texture indicates that the cement paste connects strongly to the aggregate matrix, and the reacted component fills the pores between the aggregates [69]. 

**Figure 10:**
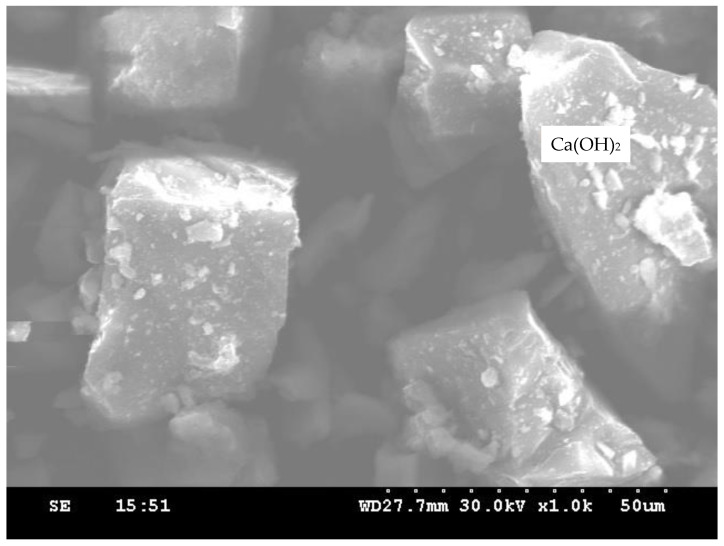
SEM image of control concrete.

**Figure 11:**
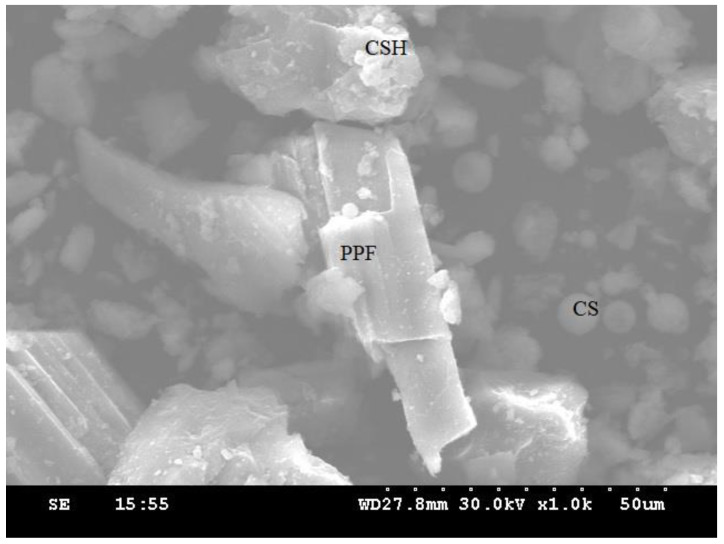
SEM image of PPF concrete.

**Figure 12:**
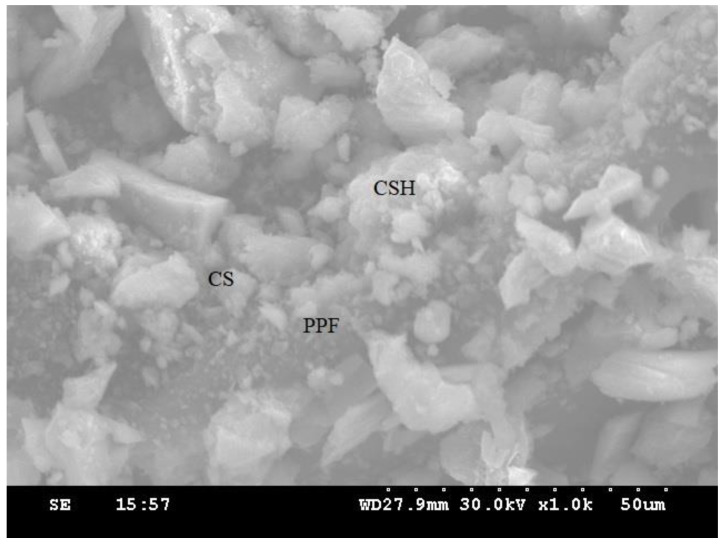
Microstructure of C20P3 composition.

Figure 10 shows the scanning electron microscope (SEM) images of the control concrete, whereas Figure 11 shows PPF concrete. A drastic change can be observed in CSH gel formation and reduction of Ca(OH)_2_. Figure 12 shows the microstructure of the optimum mix proportion obtained in this study containing 20% copper slag with 0.6% PPF. The control concrete was identified with minimum CSH gel and more unhydrated components called calcium hydroxide. The microstructure of concrete containing 20% copper slag showed moderate pozzolanic activity through the reduction of Ca(OH)_2_ components.

The PPF was surrounded by reacted components, evidenced by the superior pozzolanic activity. Comparing the control concrete and concrete containing 40%, not having any unreacted Ca(OH)_2_ is the principal reason for the increase in strength and durability. Furthermore, it accelerates the hydration of cement-containing copper slag filling the pores and resulting in a dense microstructure [70]. The microstructure of 60% replacement of copper slag in concrete evidenced some Ca(OH)_2_ components. PPF surrounded by the hydrated products ensures proper filling of minor gaps between the fibres. The round particle shape represents the copper slag, and the lean particles illustrate the PPF present in concrete. SEM images depict that unreacted Ca(OH)_2_ present in the control concrete gradually disappears in successive replacement levels of copper slag. More CSH gel formation and fewer Ca(OH)_2_ parts are identified through those images; hence the optimum replacement is 40% of CS and 0.6% of PPF. However, for up to an 80% replacement, the impact resistance of concrete does not fall below that of the control concrete.

### 4.7. Regression Analysis 

**Figure 13:**
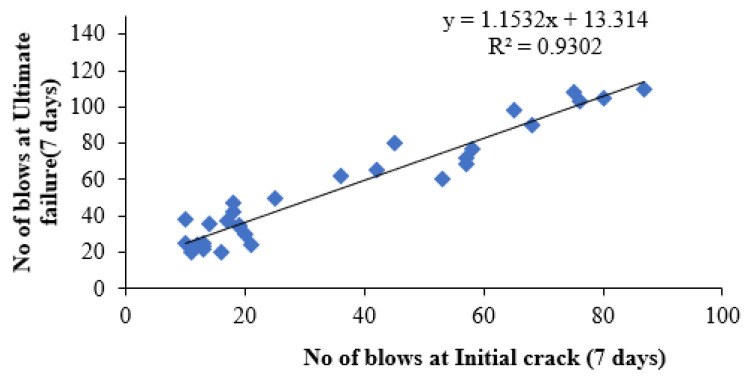
Prediction of ultimate impact resistance at 7 days.

**Figure 14:**
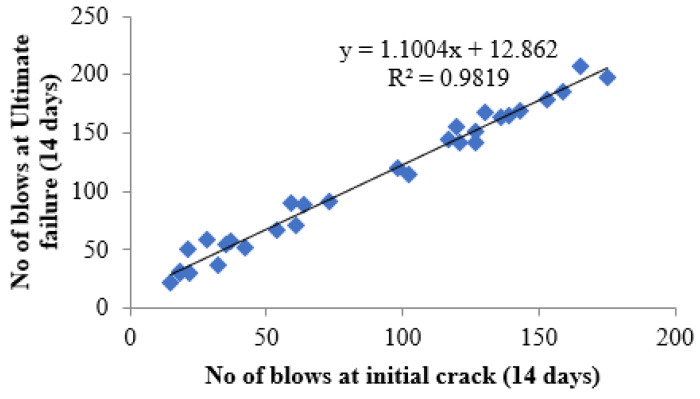
Prediction of ultimate impact resistance at 14 days.

**Figure 15:**
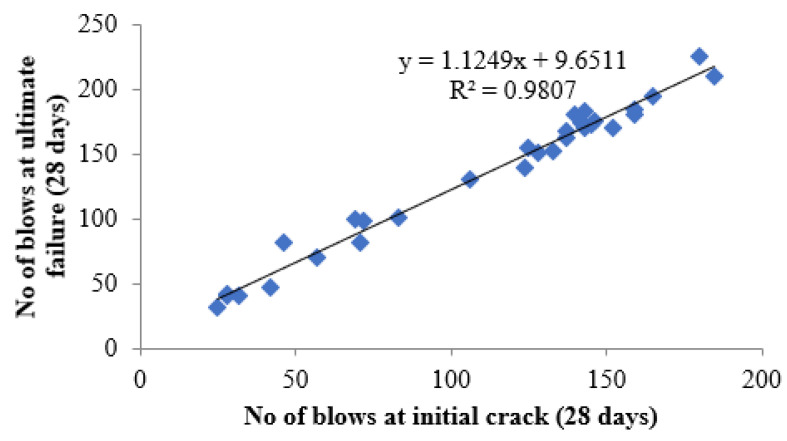
Prediction of ultimate impact resistance at 28 days.

The linear relationship between the initial cracking and ultimate failure at 7, 14 and 28 days was derived by regression analysis to analyse the interdependency of the experimental results. A strong linear relationship was proven among the N2 and N1 values. The relationships predict the number of impacts at the ultimate failure of regression coefficients 0.9302, 0.9819 and 0.9807, respectively, at 7, 14 and 28 days. Testing with more impact at the cracking level requires more hits to fail. Fibrillated PPF acts as a thread to bond the cement matrix with sharp-edged CS particles with a high specific surface area, increasing concrete’s impact resistance. PPF does not affect compressive strength, but it does increase ductility and energy absorption capacity [71]. The predicted equations are given in Equations (2)–(4), and similar to the Equation (5) derived in previous literature of polypropylene fibre reinforced concrete [72]. Figure 13, Figure 14 and Figure 15 depict a linear trend line equation with regression co-efficient at first crack and failure.

N2 = 1.1532 N1 + 13.314(2)

N2 = 1.1004 N1 + 12.862(3)

N2 = 1.1249 N1 + 9.6511(4)

N2 = 1.03 N1 + 12.96(5)

### 4.8. Reliability Analysis Using Weibull Distribution

Drop weight impact testing was performed according to ACI Committee 544 and showed significant variations in result data. Identifying the first crack is difficult since it can occur in any direction and the width of the initial crack is very small and difficult to spot it visually. The number of blows is the measure of impact resistance but the number of blows may vary according to the hardened material, such as coarse aggregate or smooth cement matrix below the point of application of impact loading. The test depends upon the manual operation of holding the mass of the hammer. Most of the crack patterns propagate along three mutual perpendicular directions from the point of application of loading. However, some specimens show different patterns and some smooth surfaces subjected to testing cause variability in crack paths [73]. The researchers employed various statistical tools to overcome the variations in the experimental test results. Normal distribution is a common tool adopted in statistical analysis but the impact test results employed with normal distribution expressed poor fitness with 95% confidence level [72]. Weibull distribution is a reliability analysis tool pack developed by Wallodi Weibull [74]. Previous studies on drop weight impact test have performed two-parameter Weibull distribution and it was found to be more adaptable and thus suggested by the researchers. It allows the data to fit more flexibly within the function. 

Weibull distribution reliability analysis accepts reliability analysis for fatigue analysis and impact testing [75]. The two-parameter Weibull distribution depends upon the shape parameter (α) and scale parameter (β), which critically influence the reliability analysis results. The probability density function and cumulative probability function are given in Equations (6) and (7), respectively, in which *t* represents the numbers of random variables between N1 and N2, *t*_0_ is the location variable where *t* ≥ *t*_0_, α > 0 and β ≥ *t*_0_.

(6) $$F\left(p\right)=\frac{\alpha }{m-t_{0}}{\left[\frac{t-t_{0}}{\beta -t_{0}}\right]}^{\alpha -1}\exp\left[-{\left(\frac{t-t_{0}}{\beta -t_{0}}\right)}^{\beta }\right]$$

(7) $$F\left(c\right)=1-\exp\left[-{\left(\frac{t-t_{0}}{\beta -t_{0}}\right)}^{\alpha }\right]$$

Equation (8) represents the probability survivorship function.

F_(PS)_ = 1 − F(c) (8)

To define the minimum life of brittle concrete composite under impact and fatigue loading, the *t*_0_ function is assumed to be zero. According to the minimum life the equation is revised to obtain revised survivorship probability (F_RSP_) as given in Equation (9).

(9) $$F_{\left(\mathrm{RSP}\right)}=\exp\left[-{\left(\frac{t}{\beta }\right)}^{\alpha }\right]$$

Taking natural log on both sides of Equation (9) twice, the following Equation (10) was obtained.

(10) $$\ln\left[\ln\left(\frac{1}{F_{\mathrm{RSP}}}\right)\right]=\alpha \ln\left(t\right)-\alpha \ln\left(\beta \right)$$

Equation (10) can be separated as individual component of a linear equation consisting of slope and intercept Y = ln$\left[\ln\left(\frac{1}{F_{\mathrm{RSP}}}\right)\right]$; X = ln(t); C = αln(β), which represents Y = α X – C.

The failure probability of each experimental result was substituted to the empirically derived survivorship function F_(S)_ known as Benard’s approximation [76] given in Equation (11).

(11) $$F_{\left(S\right)}=\frac{x-0.3}{n+0.4}$$

where *x* is the consecutive serial number of results arranged in ascending order. *n* is the total number of sample points. The graph was plotted using the data set between Y = ln$\left[\ln\left(\frac{1}{F_{\mathrm{RSP}}}\right)\right]$ as ordinated and ln(t) as abscissa. The points are conned with a linear trendline from which α and β were determined by calculating the intercepts. The impact blows N1_(R)_ or N2 _(R)_ based on reliability probability can be determined using the formula given in Equation (12).

(12) $$N1_{\left(R\right)}=\alpha \;\left(-\ln{\left(R\right)}^{\frac{1}{\beta }}\right)$$

Weibull distribution reliability analyses were executed using Microsoft Excel. The following steps are adopted to obtain the percentage of reliability on impacts obtained through the drop weight impact resistance test.

**Figure 16:**
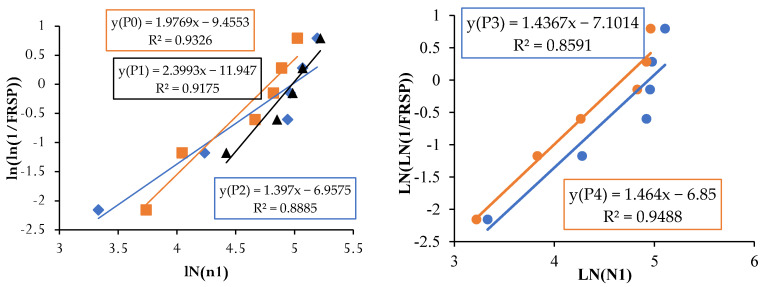
Weibull distribution for P0, P1, P2, P3 and P4 mixes at initial crack on 28 days.

**Figure 17:**
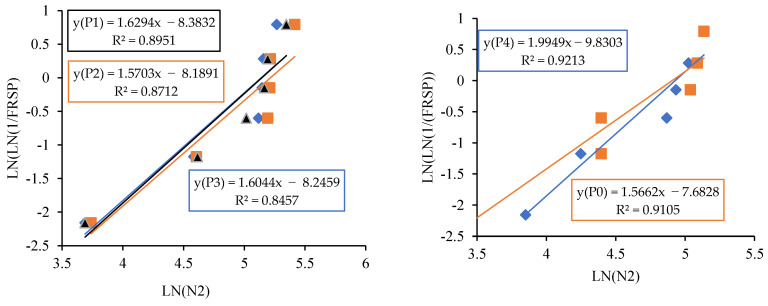
Weibull distribution for P0, P1, P2, P3 and P4 mixes at failure on 28 days.

**Figure 18:**
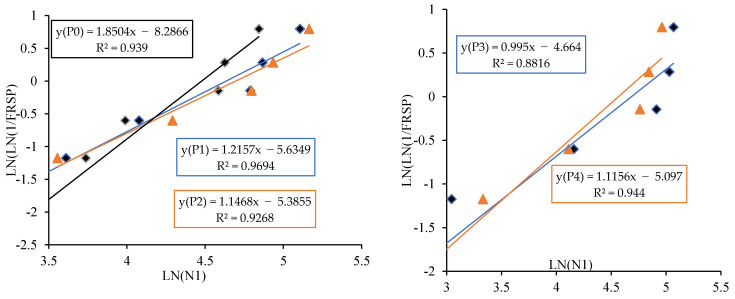
Weibull distribution for P0, P1, P2, P3 and P4 mixes at initial crack on 14 days.

**Figure 19:**
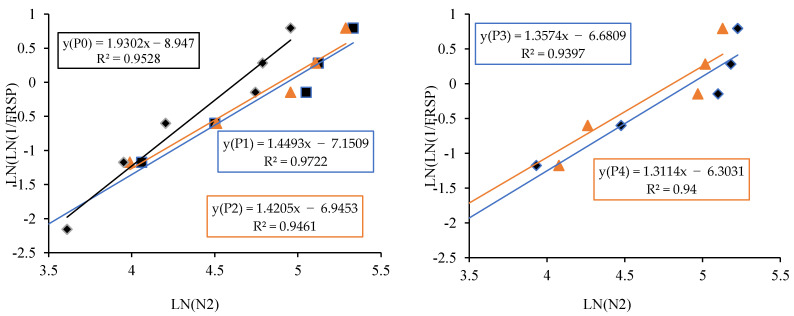
Weibull distribution for P0, P1, P2, P3 and P4 mixes at failure on 14 days.

**Figure 20:**
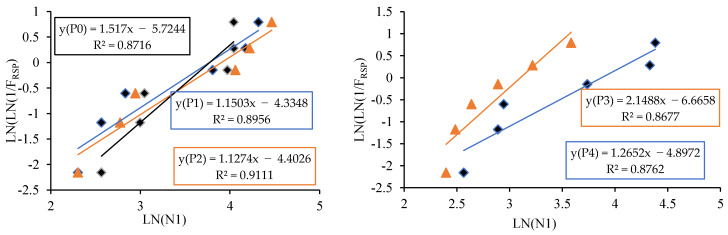
Weibull distribution for P0, P1, P2, P3 and P4 mixes at initial crack on 7 days.

**Figure 21:**
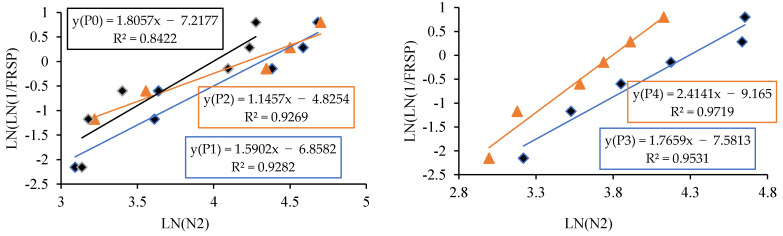
Weibull distribution for P0, P1, P2, P3 and P4 mixes at ultimate on 7 days.

- The experimental observation includes the number of impacts at cracking and failure stage on 7, 14 and 28 days. The results are grouped into P0 (0.0% PPF), P1 (0.2% PPF), P2 (0.4% PPF), P3 (0.6% PPF) and P4 (0.8% PPF).
- The number of blows in each group was sorted in ascending order and the serial number was assigned from 1 to x where x is the respective serial number.
- The failure probability F_(RSP)_ was calculated using the formula given in Equation (9).
- The natural logarithm value of N1 or N2 was determined.
- The value of equation ln$\left[\ln\left(\frac{1}{F_{\mathrm{RSP}}}\right)\right]$ was calculated.
- The graph was drawn taking ln (N1 or N2) as *x* axis and ln$\left[\ln\left(\frac{1}{F_{\mathrm{RSP}}}\right)\right]$ as *y* axis.
- The points in the graph were picked to obtain a linear trendline with equation and R^2^ value on the graph. The equation is used to obtain the value of slope and intercept C. Figure 13, Figure 14, Figure 15, Figure 16, Figure 17 and Figure 18 illustrate the linear trendline and its equation including the coefficient of regression for all batches of PPF.
- The slope of the trendline is taken as *β*.
- The intercept C = αln(β). Using the value of *β* and intercept C the value of α was calculated. Table 6 lists the shape parameter, scale parameter and regression coefficient from the trendline for different groups of PPF volume fraction, such as 0.2%, 0.4%, 0.6% and 0.8%.
- The Weibull cumulative distribution was performed using the Weibull formula. Dist (N1 or N2, α, *β*, FALSE).
- Reliability probability (R) was calculated by subtracting Revised Survivorship Probability (F_RSP_) from 1.
- The impact strength of concrete in terms of N1 and N2 was determined using the reliability probability as given in Equation (12). The graphs were drawn between the reliability coefficient and the number of blows which are shown in Figure 16, Figure 17, Figure 18, Figure 19, Figure 20 and Figure 21.

**Figure 22:**
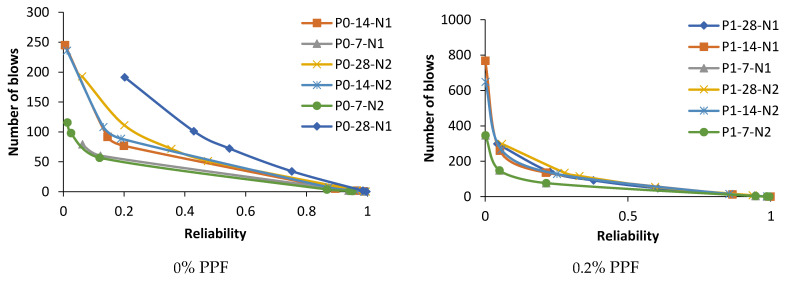

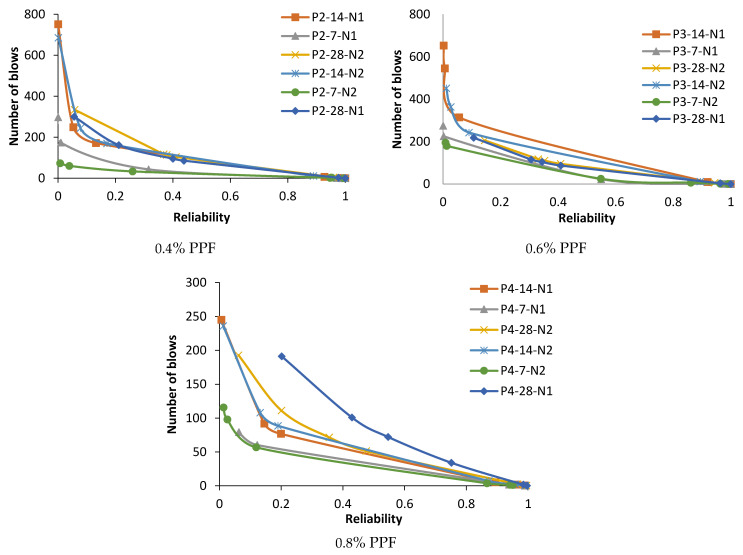
Reliability curves for P0, P1, P2, P3 and P4 mixes.

The minimum regression coefficient is 0.8422 and the maximum coefficient of determination is 0.9775. The data set fitted to the linear trendline shows the regression coefficient greater than 0.8 evidencing a good prediction leading to an accurate Weibull distribution [77,78]. The number of blows observed from the reliability curves at initial crack were 12, 80 and 100 at 7, 14 and 28 days for C0P3. For the same mix, 20, 103 and 135 are the number of blows at ultimate failure on 7, 14 and 28 days. For the next proportion, C20P3, the impacts estimated at initial cracking were 65, 98 and 130 at 7, 14 and 28 days. For the same mix, 98, 140 and 185 are the calculated impacts at ultimate failure at 7, 14 and 28 days. The reliability curves are shown in Figure 22. Proportion C40P3 observed at initial crack was 75, 140 and 160 at 7, 14 and 28 days. For the same mix, 115, 185 and 250 are the blows calculated at ultimate failure at 7, 14 and 28 days. C60P3 observed blows at initial crack were 60, 122 and 125 at 7, 14 and 28 days. For the same mix, 83, 162 and 165 are the blows at failure state at 7, 14 and 28 days. Proportion C80P3 observed counts at initial crack were 45, 80 and 90 at 7, 14 and 28 days. For the same mix, 60, 90 and 115 are the counts at ultimate failure at 7, 14 and 28 days. The estimated counts for C100P3 at initial cracking were 7, 12 and 18 at 7, 14 and 28 days. For the same mix, at failure stage on 7, 14 and 28 days the counts were 12, 20 and 28. The reliability level for the above values is 0.8 or greater than 0.8. Weibull distribution estimates the number of blows in terms of reliability level considering the field variation of taking an average of experimental results [79,80].

### 4.9. Energy Absorption Capacity of Concrete 

**Figure 23:**
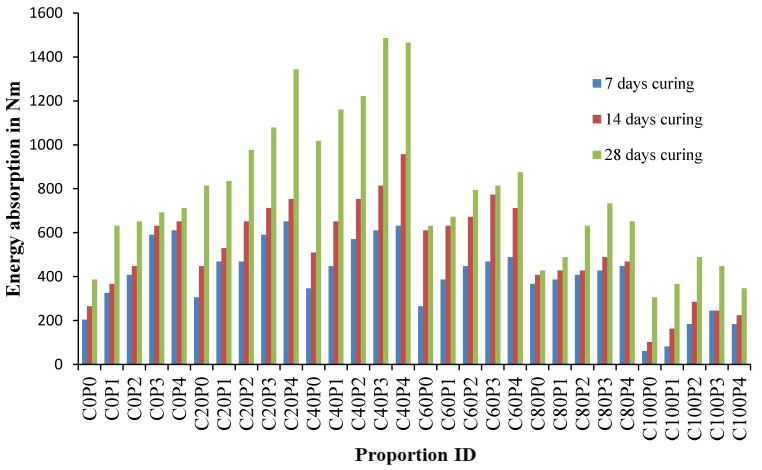
Energy absorption capacity.

Energy absorption is the amount of work done or the amount of energy stored in the material. Materials with high energy absorption are more resistant to impact loading. Fibres are primarily energy-storing materials needed explicitly in impact loading or any other accidental loading where ductility is the primary factor. From Figure 23, it is clear that the concretes which hold 20% copper slag and 40% copper slag replaced with sand perform well in absorbing the impact force. Concrete mix proportion C40 series stored high energy to resist the applied loading.

The maximum energy absorption values in the C40P3 mix are 610.61, 814.14 and 1485.81 Nm at 7, 14 and 28 days. The energy absorption values of the control concrete at 7, 14 and 28 days were 203.54 Nm, 264.6 Nm and 386.72 Nm. The corresponding increase in percentages is 199%, 207% and 284%, which is approximately the energy absorption increased two times. Previous literature stated that the energy absorption at 7, 14 and 28 days is doubled for the fibrillated fibre reinforced concrete, which was confirmed in this experimental work [81,82] The trend-line depicts that the energy absorption increases up to 40% and decreases further. However, the energy absorption within the concrete in the C100 series is higher than the control concrete at 28 days.

## 5. Conclusions

Wastes generated by various industries can be used in multiple ways in the field of solid waste management. Using alternative materials for cement and fine aggregate reduces consumption and increases natural resource conservation. The experimental part of the study includes the testing of concrete under impact loading according to ACI drop weight method and the analytical part of the study considers the variability of results obtained during experimental work and arriving at normalised values for number of blows using Weibull distribution. At 40% of copper slag replacement, the impact resistance is high at 0.8% PPF and energy absorption was a maximum at 0.6%. Hence, optimum replacement of copper slag is 40% and optimum volume fraction of polypropylene fibre is 0.6–0.8%. This research work brings the following conclusions:

− Copper slag has many potential benefits such as improving workability, increasing impact resistance and producing pozzolanic properties. Copper slag would be an exceptional substitute for fine aggregate. There is no detrimental effect detected on replacing the fine aggregate up to 80% CS. CS 40% and PPF 0.4% shows optimum replacement according to compressive strength results. The C100 group does not attain required target strength due to the free water content in the mix. The free water content is attributed to the low water absorption of CS.
− PPF in CS concrete enhances the ductility, post crack performance and increases the capacity against failure compared to the mixes without PPF. A 0.2–0.6% of PPF volume fraction shows improved performance compared to 0.8% PPF. The networking structure of bonding within the fibre concrete matrix reduces the linear and lateral expansion of cracks and hence the count at first crack itself increased. Fibrillated fibre shows higher post-cracking resistance.
− The impact count gradually increases up to 80% replacement of copper slag. In each batch of CS replacement there is an increasing trend of blows according to PPF content. Concrete containing 100% copper slag replaced with sand tolerated 35–47% less blows to break the specimen. C40P4 tolerated the highest number of blows of 82, 158 and 172 at 7, 14 and 28 days, respectively. Blows at initial crack increased from 2.66 to 2.91 times. The impact blows increase from 2.75 to 3.10 times over the control concrete at 28 days at ultimate failure.
− The hydration product increases in CS mixes compared to the control concrete can be evidenced through microstructure images. Due to the excellent pozzolanic activity of CS the amount of CSH gel formation increases with each increment of CS, and thereby a reduction of unreacted Ca(OH)_2_ is visualized in SEM images. The microstructure of concrete evidences homogeneous spread of CSH gel, pore filling effect and dense microstructure.
− Regression analysis was performed to derive a relationship between early cracking and ultimate failure. The proposed best fit of linear equations predicts the N2 values from N1 derived the regression coefficients of 0.9302, 0.9819 and 0.9807, respectively at 7, 14 and 28 days.
− Weibull distribution using shape and scale parameters is suitable for distributing the variation in number of blows at initial crack and ultimate failure. The impact blow counts observed at reliability level 0.8 or greater is significantly related to the tabulated blows during experimentation.
− The energy absorption values are doubled in the case of fibre reinforced proportions. Maximum values of energy absorption were found in the C40P3 mix, respectively, 610.61, 814.14 and 1485.81 Nm at 7, 14 and 28 days. At the same time, the energy absorption values of the control concrete at 7, 14 and 28 days were 203.54, 264.6 and 386.72 Nm, respectively.
− The outcome of this study describes the mix proportioning of CS-PPF concrete, characterization of microstructure, crack propagation during impact loading and finally, the optimum content of CS and PPF. Since copper slag is a pozzolanic material, like fly ash, the copper slag would be an essential alternative to fine aggregate in the future. Copper slag PPF concrete can be employed to produce solid blocks, hollow blocks and precast elements. This research work can be extended to other types of concrete-like high-density concretes, self-compacting concrete, etc.

## Figures and Tables

**Table 1:** Properties of materials.

Properties	Fine Aggregate	Copper Slag	Coarse Aggregate
Bulk density, kg/m^3^	1420	1750	1380
Specific gravity	2.51	3.56	2.80
Water absorption (%)	1.25	0.15	0.92
Fineness modulus	2.742	2.99	6.133

**Table 2:** Chemical composition of cement and copper slag.

Components	Cement (%)	CS (%)
SiO_2_	20.85	33.05
Al_2_O_3_	4.78	2.79
Fe_2_O_3_	3.51	53.45
CaO	63.06	6.06
MgO	2.32	1.56
SO_3_	2.48	1.89
K_2_O	0.55	0.61
Na_2_O	0.24	0.28
TiO_2_	0.25	0
Na_2_O	0.05	0.06
SiO_2_ + Al_2_O_3_ + Fe_2_O_3_ + CaO	92.2	95.35

**Table 3:** Mix proportion of the control concrete.

Constituents	Cement	Sand	Coarse Aggregate	Water
Quantity per m^3^	363 kg/m^3^	620 kg/m^3^	1343 kg/m^3^	148.8 kg/m^3^
Proportion	1	1.71	3.71	0.41

**Table 4:** Mix proportions.

S.No.	Mix ID	Cement	Fine Aggregate	Copper Slag	Coarse Aggregate	Water Content	Polypropylene Fibre
		kg/m^3^					
1	C0P0	363	620	0	1343	148.8	0
2	C0P1	363	620	0	1343	148.8	1.82
3	C0P2	363	620	0	1343	148.8	3.64
4	C0P3	363	620	0	1343	148.8	5.46
5	C0P4	363	620	0	1343	148.8	7.28
6	C20P0	363	495	177	1343	148.8	0
7	C20P1	363	495	177	1343	148.8	1.82
8	C20P2	363	494	177	1343	148.8	3.64
9	C20P3	363	493	176	1343	148.8	5.46
10	C20P4	363	492	176	1343	148.8	7.28
11	C40P0	363	371	354	1343	148.8	0
12	C40P1	363	371	354	1343	148.8	1.82
13	C40P2	363	371	354	1343	148.8	3.64
14	C40P3	363	370	353	1343	148.8	5.46
15	C40P4	363	369	352	1343	148.8	7.28
16	C60P0	363	247	531	1343	148.8	0
17	C60P1	363	247	531	1343	148.8	1.82
18	C60P2	363	247	530	1343	148.8	3.64
19	C60P3	363	246	529	1343	148.8	5.46
20	C60P4	363	246	528	1343	148.8	7.28
21	C80P0	363	124	708	1343	148.8	0
22	C80P1	363	124	708	1343	148.8	1.82
23	C80P2	363	124	707	1343	148.8	3.64
24	C80P3	363	123	705	1343	148.8	5.46
25	C80P4	363	123	704	1343	148.8	7.28
26	C100P0	363	620	0	1343	148.8	0
27	C100P1	363	620	0	1343	148.8	1.82
28	C100P2	363	620	0	1343	148.8	3.64
29	C100P3	363	620	0	1343	148.8	5.46
30	C100P4	363	620	0	1343	148.8	7.28

C—copper slag (CS), P—polypropylene fibre (PPF), P0—0.0% PPF, P1—0.2% PPF, P2—0.4% PPF, P3—0.6% PPF, P4—0.8% PPF, C20—20% CS, C40—40% CS, C60—60% CS, C80—80% CS, C100—100% CS.

**Table 5:** Number of blows and energy absorption values.

Mix ID	N1	N2	N2 − N1	E	N1	N2	N2 − N1	E	N1	N2	N2 − N1	E
	7 Days				14 Days				28 Days			
C0P0	13	23	10	203.54	64	77	13	264.6	84	103	19	386.72
C0P1	19	35	16	325.66	78	96	18	366.36	109	140	31	630.96
C0P2	17	37	20	407.07	83	105	22	447.78	112	144	32	651.31
C0P3	18	47	29	590.25	92	123	31	630.96	125	159	34	692.02
C0P4	25	55	30	610.61	101	133	32	651.31	132	167	35	712.38
C20P0	57	72	15	305.3	85	107	22	447.78	109	149	40	814.14
C20P1	66	89	23	468.13	92	118	26	529.19	116	157	41	834.5
C20P2	75	98	23	468.13	106	138	32	651.31	121	169	48	976.97
C20P3	76	105	29	590.25	110	145	35	712.38	124	177	53	1078.74
C20P4	80	112	32	651.31	117	154	37	753.08	126	192	66	1343.34
C40P0	57	74	17	346.01	127	152	25	508.84	133	183	50	1017.68
C40P1	68	90	22	447.78	138	170	32	651.31	146	203	57	1160.15
C40P2	65	93	28	569.9	146	183	37	753.08	161	221	60	1221.22
C40P3	80	110	30	610.61	152	192	40	814.14	173	246	73	1485.81
C40P4	82	113	31	630.96	158	205	47	956.62	172	244	72	1465.46
C60P0	60	73	13	264.6	102	132	30	610.61	124	155	31	630.96
C60P1	61	80	19	386.72	121	152	31	630.96	128	161	33	671.67
C60P2	65	87	22	447.78	127	160	33	671.67	143	182	39	793.79
C60P3	69	92	23	468.13	137	175	38	773.44	145	185	40	814.14
C60P4	74	98	24	488.49	127	162	35	712.38	151	194	43	875.2
C80P0	36	54	18	366.36	76	96	20	407.07	90	111	21	427.43
C80P1	39	58	19	386.72	81	102	21	427.43	93	117	24	488.49
C80P2	48	68	20	407.07	89	110	21	427.43	108	139	31	630.96
C80P3	56	77	21	427.43	94	118	24	488.49	112	148	36	732.73
C80P4	56	78	22	447.78	94	117	23	468.13	109	141	32	651.31
C100P0	21	24	3	61.06	36	41	5	101.77	42	57	15	305.3
C100P1	16	20	4	81.41	26	34	8	162.83	32	50	18	366.36
C100P2	13	22	9	183.18	22	36	14	284.95	28	52	24	488.49
C100P3	13	25	12	244.24	22	34	12	244.24	28	50	22	447.78
C100P4	13	20	9	183.18	19	30	11	223.89	25	42	17	346.01

N1—number of blows at first crack, N2—number of blows at failure, E—energy absorption in Nm, E = (N2 − N1) m g h, m—mass of hammer in kg, h—height of fall in m, g = 9.81 kN/m^3^.

**Table 6:** Weibull distribution parameters and regression coefficients.

Mix Identification	Curing Period	Number Blows	Scale Parameter (β)	Intercept	Shape Parameter (α)	Regression Coefficient (R^2^)
P0 (0.0% PPF)	7	N1	1.517	−5.724	43.532	0.8716
		N2	1.805	−7.217	49.432	0.8422
	14	N1	1.850	−8.286	88.086	0.939
		N2	1.93	−8.947	103.056	0.9528
	28	N1	1.976	−9.455	119.440	0.9326
		N2	1.995	−9.830	138.063	0.9213
P1 (0.2% PPF)	7	N1	1.407	−5.967	69.517	0.915
		N2	1.127	−4.4026	49.654	0.9111
	14	N1	1.258	−5.92	110.276	0.9625
		N2	1.438	−7.0336	132.937	0.9775
	28	N1	1.556	−7.769	146.930	0.9087
		N2	1.624	−8.383	171.564	0.8951
P2 (0.4% PPF)	7	N1	1.15	−4.334	43.311	0.8956
		N2	1.59	−6.858	74.648	0.9282
	14	N1	1.215	−5.634	103.039	0.9694
		N2	1.449	−7.15	138.939	0.9722
	28	N1	1.397	−6.957	145.520	0.8885
		N2	1.570	−8.189	184.010	0.8712
P3 (0.6% PPF)	7	N1	1.265	−4.897	47.975	0.8762
		N2	1.765	−7.581	73.197	0.9531
	14	N1	0.995	−4.664	108.574	0.8816
		N2	1.357	−6.68	137.254	0.9397
	28	N1	1.437	−7.101	140.160	0.8591
		N2	1.604	−8.245	170.639	0.8457
P4 (0.8% PPF)	7	N1	2.148	−6.665	22.244	0.8677
		N2	2.414	−9.165	44.54	0.9719
	14	N1	1.115	−5.097	96.432	0.944
		N2	1.311	−6.303	122.289	0.94
	28	N1	1.464	−6.850	107.650	0.9488
		N2	1.566	−7.682	135.013	0.9105

## Data Availability

Data sharing not applicable.
